# Pathogen detection and microbial community characterization in clinically symptomatic pigs from commercial farms

**DOI:** 10.3389/fvets.2026.1947134

**Published:** 2026-10-01

**Authors:** Se Hui Park, Doo Sung Cheon, Tae Jin Cho, Sun Ae Kim

**Affiliations:** 1 Department of Food Science and Biotechnology, Ewha Womans University, Seoul, Republic of Korea; 2 Postbio Inc., Namyangju, Republic of Korea; 3 Department of Food and Biotechnology, College of Science and Technology, Korea University, Sejong-si, Republic of Korea; 4 Department of Food Regulatory Science, College of Science and Technology, Korea University, Sejong-si, Republic of Korea

**Keywords:** 16S rRNA gene sequencing, clinically symptomatic pigs, enteric microbiota, molecular diagnostics, respiratory microbiota, swine disease pathogens

## Abstract

Effective swine disease control requires continuous monitoring of major pathogens and a comprehensive understanding of microbial community dynamics under disease conditions. Here, we collected lung, oral fluid, and fecal samples from clinically symptomatic pigs on commercial farms in South Korea. Molecular diagnostics targeting major respiratory and enteric pathogens were performed to assess infection status, alongside 16S rRNA gene sequencing to simultaneously characterize respiratory and enteric microbiota. Most respiratory pathogens were detected at higher rates in oral fluid than in lung samples. *Pasteurella multocida* was the most frequently detected pathogen overall, while porcine reproductive and respiratory syndrome virus (PRRSV) showed the highest detection rate in lung samples. Among enteric pathogens, rotavirus showed the highest detection rate. Microbiome profiling revealed that lung samples had the lowest species diversity, while distinct microbial community structures were observed among lung, oral fluid, and fecal samples. Prevotellaceae-associated genera were dominant in fecal samples. The lung microbiota was characterized by dominance of a limited number of taxa, including *Escherichia*–*Shigella* and *Actinobacillus,* whereas oral fluid exhibited a more evenly distributed microbial composition enriched in *Moraxella* and *Streptococcus*. Differentially abundant taxa and contrasting predicted functional profiles were identified between *Clostridium perfringens-*positive and -negative groups, indicating a potential association between *Clostridium perfringens* presence and gut microbial community composition. This study integrates molecular diagnostics with microbiome profiling in clinically diseased pigs under field conditions. These findings provide field-based evidence to support microbiome-informed monitoring and interpretation of pathogen-associated changes in swine production systems.

## 1 Introduction

Globally, pigs are affected by a diverse array of viral and bacterial diseases, which lead to reduced growth rates, increased mortality, elevated treatment costs, greater antibiotic usage, and significant economic losses in the swine industry (1). Respiratory diseases commonly begin with primary infections by pathogens such as porcine circovirus (PCV2, PCV3) and porcine reproductive and respiratory syndrome virus (PRRSV), followed by secondary bacterial infections involving *Pasteurella multocida* and *Actinobacillus pleuropneumoniae*, culminating in porcine respiratory disease complex (PRDC) (2, 3). Enteric diseases are primarily associated with pathogenic *Escherichia coli*, rotavirus, and *Clostridium perfringens* (4, 5).

The rapid spread of these pathogens is facilitated by intensive swine production systems, characterized by group housing, close animal contact, and shared feeding and watering systems, leading to rapid transmission within farms (6). Consequently, rapid and accurate pathogen detection at early stages of infection is critical for effective disease control (7). Early diagnostic information supports timely implementation of on-farm interventions, including enhanced biosecurity (e.g., quarantine, isolation of affected animals, movement restrictions, and disinfection) and targeted treatment strategies, thereby mitigating transmission and reducing economic losses (8). From a One Health perspective, these measures also help minimize pathogen dissemination beyond farm boundaries, lower the risk of environmental contamination and zoonotic transmission, and promote judicious antimicrobial use (9). Accordingly, molecular diagnostic methods with high sensitivity and specificity are widely adopted in clinical settings to rapidly detect and quantify pathogen genetic material, enabling effective monitoring of disease status and infection dynamics (10).

Importantly, the onset and progression of disease are influenced not only by individual pathogens but also by complex interactions among multiple microorganisms. The microbiota plays a crucial role in modulating host immune responses and influencing pathogen colonization (11). Thus, a comprehensive understanding of disease states requires an integrated approach that considers both pathogen presence and the structure of the microbial community (11, 12). However, studies simultaneously investigating pathogen detection and microbiome composition in clinically affected pigs under commercial farming conditions remain scarce.

In this study, we collected lung (lower respiratory tract), oral fluid (upper respiratory tract), and fecal samples from clinically symptomatic pigs on commercial farms in South Korea. Multiplex real-time quantitative polymerase chain reaction (qPCR) and one-step reverse transcription qPCR (RT-qPCR) assays were performed to detect major swine pathogens and to assess infection status under field conditions. In parallel, high-throughput 16S rRNA sequencing was conducted to compare microbiota across different sample types and to characterize respiratory and enteric microbiota communities. Additionally, molecular diagnostic results were integrated with microbial community data to explore potential associations between pathogen presence and microbial community structure.

## 2 Materials and methods

### 2.1 Ethics statement

All samples were clinical specimens submitted to a veterinary diagnostic laboratory accredited by the Animal and Plant Quarantine Agency (APQA) for routine livestock disease diagnosis. Under the Korean Laboratory Animal Act, animal testing is defined as experiments or scientific procedures conducted on laboratory animals for scientific purposes. As these specimens were obtained for diagnostic purposes and no experimental procedures on live animals were performed specifically for this study, no approval was required.

### 2.2 Sample collection

All samples in this study were clinical specimens submitted to a veterinary diagnostic laboratory (Postbio Inc., Namyangju, South Korea) accredited by APQA for livestock disease diagnostics. The samples were collected from pigs exhibiting disease symptoms in South Korea between October 2023 and December 2024. Fecal samples (*n* = 79) were obtained from pigs with enteric disease symptoms at 34 farms. Respiratory samples were collected from pigs presenting with typical respiratory symptoms: oral fluid samples (upper respiratory tract, *n* = 67) were collected from 17 farms as described by Prickett et al. (13), and lung samples (lower respiratory tract, *n* = 33) were obtained from 21 farms. Lung samples were collected post-mortem during necropsy from grossly affected lung areas. To minimize surface contamination, the pleural surface was seared with a flame-heated sterile spatula and then incised. The exposed internal lung tissue was sampled using a sterile swab. The distribution of samples across farms is provided in Supplementary Table S1.

### 2.3 DNA extraction and molecular diagnostic testing

DNA and RNA were extracted using the APDx™ Viral NA Kit (APBio, Namyangju, South Korea) according to the manufacturer’s instructions and stored at −80 °C for analysis. Multiplex qPCR/RT-qPCR assays were performed on clinical samples to qualitatively detect pathogens and toxin-associated genes associated with swine enteric and respiratory diseases (Supplementary Table S2). Each reaction was conducted in a final volume of 20 μL, consisting of 15 μL of reaction mixture and 5 μL of template DNA or RNA. For DNA targets (multiplex qPCR), the reaction conditions included an initial denaturation step at 95 °C for 10 min, followed by 45 cycles of denaturation at 95 °C for 10 s, and combined annealing and extension at 60 °C for 30 s. For RNA targets (one-step RT-qPCR), an additional reverse transcription step at 50 °C for 15 min was performed before the initial denaturation, and amplification was then performed under the same cycling conditions. Plasmid DNA and nuclease-free water were included as positive and negative controls, respectively, in each assay run. An internal control was included in each reaction. Samples with target Ct values <35 were considered positive. The analytical limit of detection was approximately 100 copies per reaction.

### 2.4 Library preparation and 16S rRNA gene sequencing

The DNA samples comprised 33 lung, 67 oral fluid, and 79 fecal samples. Library preparation followed established protocols (14–17). The V4 region of the 16S rRNA gene was amplified using dual-index primers, 515F (5′-GTGCCAGCMGCCGCGGTAA-3′) and 806R (5′-GGACTACHVGGGTWTCTAAT-3′), following the method of Kozich et al. (18). PCR was performed in a final volume of 20 μL using AccuPrime™ Pfx SuperMix (Thermo Fisher Scientific, Waltham, MA, USA). Amplification consisted of initial denaturation at 95 °C for 2 min, followed by 35 cycles of 95 °C for 30 s, 55 °C for 30 s, and 77 °C for 1 min, with a final extension at 72 °C for 5 min. PCR products were analyzed by gel electrophoresis, and amplification results were visualized using the Gel Doc XR c150 system (Bio-Rad, Hercules, CA, USA). Thirteen lung samples were excluded at this stage due to the absence of PCR amplification bands. Nuclease-free water was included as a negative control in each PCR run, and no amplification was observed in any negative control. Extraction blanks were not included in this study. Amplified PCR products were normalized using the SequalPrep™ Normalization Plate Kit (Applied Biosystems, Waltham, MA, USA) according to the manufacturer’s instructions. Quality was assessed by measuring the concentration of normalized products using Qubit 4.0 (Thermo Fisher Scientific). Subsequently, 1 μL of each product was pooled into microcentrifuge tubes for library preparation. Quantitative and qualitative assessment of pooled libraries was performed by qPCR (QuantStudio 1, Applied Biosystems) using the KAPA Library Quantification Kit (Kapa Biosystems, Wilmington, NC, USA).

Pooled libraries and PhiX control v3 (Illumina, San Diego, CA, USA) were each mixed with 0.2 N NaOH and diluted with HT1 buffer to obtain a final concentration of 6 pM. The final loading library was prepared by combining 480 μL of the denatured library with 120 μL of the denatured PhiX control, resulting in a final PhiX proportion of 20% (v/v). Sequencing was performed on the Illumina MiSeq platform using the MiSeq® Reagent Kit v2 (500-cycles, paired-end).

### 2.5 Sequencing data processing

The generated FASTQ files were processed using QIIME 2 (version 2023.2). Demultiplexed paired-end reads were denoised using the Divisive Amplicon Denoising Algorithm 2 (DADA2, version 1.26.0) pipeline, which removed low-quality bases, PhiX, and chimeric sequences. Forward and reverse reads were truncated at 250 bp and 231 bp, respectively, with no left trimming applied. Reads were filtered using a maximum expected error threshold of 2.0 for both forward and reverse reads, and chimeric sequences were identified using the consensus method. Amplicon sequence variants (ASVs) were taxonomically classified using the q2-feature-classifier plugin with the classify-sklearn method (19). Classification was based on a pre-trained naive Bayes classifier built on the SILVA 138 SSU NR99 reference database (silva-138-99-nb-classifier.qza), with a confidence threshold of 0.7. ASVs assigned to chloroplasts, mitochondria, and eukaryota were excluded. One lung sample and one oral fluid sample were excluded due to low sequencing depth (<1,000 reads after quality filtering), yielding a final dataset of 19 lung, 66 oral fluid, and 79 fecal samples for microbiota analysis.

### 2.6 Statistical analysis

Odds ratios for pathogen co-occurrence were calculated from 2 × 2 contingency tables within each sample type (lung, oral fluid, and fecal samples) using R (version 4.5.1). Statistical significance was assessed using two-sided Fisher’s exact tests based on sample-level binary detection data (positive/negative). Alpha diversity at the genus level (Chao1 richness and Shannon diversity index) was assessed using Kruskal–Wallis and Mann–Whitney tests. Beta diversity, based on Bray–Curtis dissimilarity, was visualized using principal coordinates analysis (PCoA) plot, and statistically tested with PERMANOVA and PERMDISP. Linear discriminant analysis effect size (LEfSe) analysis was used to identify bacteria enriched in each group. MicrobiomeAnalyst (20) was used for alpha and beta diversity and LEfSe analyses. Functional profiles of bacterial communities were predicted using Phylogenetic Investigation of Communities by Reconstruction of Unobserved States 2 (PICRUSt2, version 2.5.2) (21). Statistical analysis and visualization of predicted functional profiles were performed using STAMP software (version 2.1.3) (22). Differences between groups were evaluated using a two-sided Welch’s t-test with a 95% confidence interval (CI). For all analyses, *p*-values were adjusted for multiple comparisons using the Benjamini–Hochberg false discovery rate (FDR) procedure. The criterion of significance was defined as an FDR-adjusted *p*-value < 0.05.

The number of samples contributed by individual farms varied. Therefore, sensitivity analyses accounting for potential farm-level clustering were performed using permutations restricted within farms. For alpha diversity, the Mann–Whitney U statistic was used as the test statistic, and its null distribution was generated by permuting pathogen detection status within farms. For beta diversity, PERMANOVA based on Bray–Curtis dissimilarity was performed with permutations similarly restricted within farms. Both analyses were conducted using 999 permutations. The analyses were also repeated after restricting the dataset to farms that contributed at least one pathogen-positive and one pathogen-negative sample. All restricted permutation analyses were performed in R using the vegan and permute packages. Because these analyses were performed as sensitivity analyses to assess the robustness of the primary comparisons rather than as independent hypothesis tests, the permutation-derived *p*-values were not subjected to an additional multiplicity adjustment.

## 3 Results

### 3.1 Pathogen detection and co-occurrence in lung, oral fluid, and fecal samples

Table 1 summarizes the detection rates of pathogens across distinct sample types. Respiratory samples (lung, *n* = 33; oral fluid, *n* = 67) were analyzed for respiratory-associated pathogens, including PRRSV, PCV2, PCV3, *A. pleuropneumoniae*, and *P. multocida*. Fecal samples (*n* = 79) were assessed for enteric pathogens: rotavirus, pathogenic *E. coli*, and *C. perfringens*. Detection rates differed between lung and oral fluid samples depending on the specific pathogen. In lung samples, PRRSV showed the highest detection rate (60.6%), followed by PCV2 (39.4%), *P. multocida* (18.2%), PCV3 (15.2%), and *A. pleuropneumoniae* (6.1%). Detection rates in oral fluid were generally higher than in lung samples, with *P. multocida* most frequently detected (76.1%), followed by PRRSV (53.7%), PCV3 (47.8%), PCV2 (43.3%), and *A. pleuropneumoniae* (32.8%). Among enteric pathogens in fecal samples, rotavirus showed the highest detection rate (43.0%), followed by *C. perfringens* (30.4%) and pathogenic *E. coli* (25.3%). All pairwise combinations of pathogens within each sample type were tested for co-occurrence. No statistically significant co-occurrences were observed in lung, oral fluid, or fecal samples (Supplementary Table S3).

**Table 1:** Detection rates of respiratory pathogens in lung and oral fluid samples, and enteric pathogens in fecal samples.

Pathogen group	Diagnostic pathogen	Detection rate % (Number of positives)		
		Lung (*n* = 33)	Oral fluid (*n* = 67)	Feces (*n* = 79)
Respiratory pathogens	PRRSV	60.6% (20)	53.7% (36)	N/A
	PCV2	39.4% (13)	43.3% (29)	N/A
	PCV3	15.2% (5)	47.8% (32)	N/A
	*Actinobacillus pleuropneumoniae*	6.1% (2)	32.8% (22)	N/A
	*Pasteurella multocida*	18.2% (6)	76.1% (51)	N/A
Enteric pathogens	Rotavirus	N/A	N/A	43.0% (34)
	Pathogenic *Escherichia coli*	N/A	N/A	25.3% (20)
	*Clostridium perfringens*	N/A	N/A	30.4% (24)

N/A, not applicable.

### 3.2 Comparison of microbiota between lung, oral fluid, and fecal samples

Sequencing quality was assessed based on the run metrics generated by the Illumina MiSeq platform, with the percentage of base calls above Q30 (88.82%) and a low error rate (2.11%). In total, 164 samples were included in the microbiota analysis, comprising lung (*n* = 19), oral fluid (*n* = 66), and fecal (*n* = 79) specimens.

Alpha diversity was assessed among lung, oral fluid, and fecal samples using the Chao1 and Shannon indices (Figure 1A). The Chao1 index reflects species richness, while the Shannon index accounts for both richness and evenness (23). At the genus level, significant overall differences among the three sample types were detected for both indices. Pairwise comparisons showed a consistent pattern across Chao1 and Shannon: lung samples (lower respiratory tract) exhibited significantly lower values than oral fluid and fecal samples (both *p* < 0.001), whereas no significant differences were observed between oral fluid and fecal samples (*p* > 0.05). Beta diversity was visualized using PCoA based on Bray–Curtis dissimilarity to compare microbial community structures among the three sample types (Figure 1B). In the PCoA plot, shorter distances between dots indicate greater compositional similarity. Microbial communities differed significantly among sample types, forming three distinct clusters (*F* = 19.476, R^2^ *=* 0.19481, *p* = 0.001). Pairwise PERMANOVA further confirmed significant differences among all sample-type pairs (Supplementary Table S4; all *p* = 0.001). The *F*-value represents the magnitude of community differences, with higher values indicating greater separation between groups: oral fluid–feces (*F* = 26.917), lung–feces (*F* = 18.045), and lung–oral fluid (*F* = 10.278). This pattern was consistent with the PCoA plot, in which the lung and oral fluid clusters partially overlapped, and the fecal cluster was clearly separated (Figure 1B). Overall, the fecal microbiota formed a distinct community structure compared to respiratory-associated samples.

**Figure 1:**
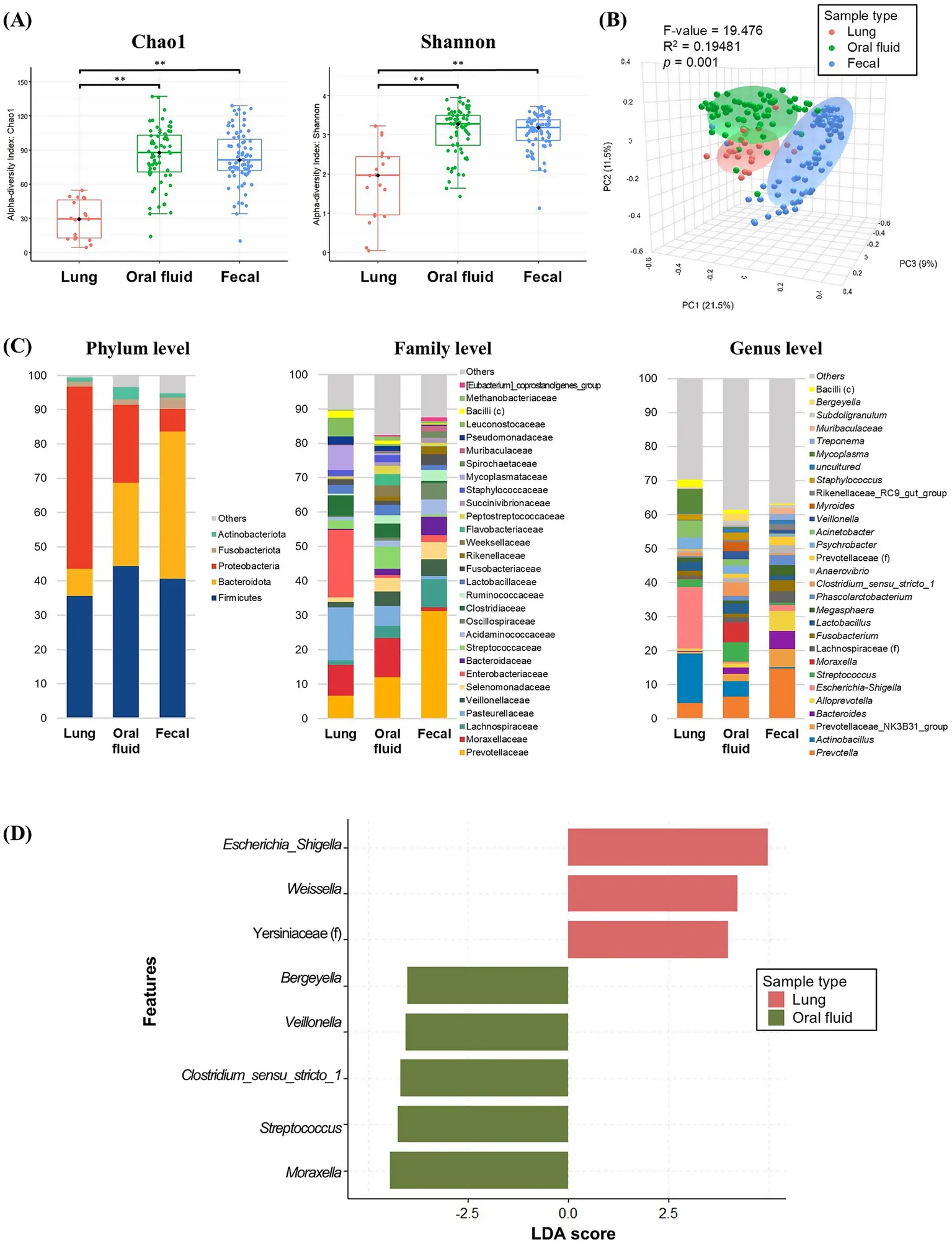
Microbial community diversity and composition across lung (*n* = 19), oral fluid (*n* = 66), and fecal samples (*n* = 79). **(A)** Alpha diversity (Chao1 and Shannon indices) compared using the Mann–Whitney and Kruskal–Wallis test. **p < 0.001. **(B)** Beta diversity visualized by principal coordinates analysis (PCoA) based on Bray–Curtis dissimilarity. **(C)** Mean relative abundance of microbial community taxa at the phylum, family, and genus levels. **(D)** Differentially abundant taxa between lung and oral fluid samples identified by linear discriminant analysis effect size (LEfSe). LEfSe analysis was performed with a linear discriminant analysis (LDA) score ≥ 3 and a false discovery rate *p*-value < 0.05.

To assess taxonomic differences among sample types, the top 5 phyla, 29 families, and 29 genera were identified, with all other taxa grouped as “Others.” Mean relative abundances for each sample type are presented in Figure 1C. Taxonomic composition diverged across multiple levels among lung, oral fluid, and fecal samples. At the phylum level, Firmicutes (40.63%) and Bacteroidota (42.97%) were predominant in feces. In contrast, Proteobacteria were relatively more abundant in respiratory samples. At the family level, Prevotellaceae (31.26%) was predominant in feces. This pattern was consistently reflected at the genus level, where *Prevotella* (14.66%), Prevotellaceae_NK3B31_group (5.33%), and *Alloprevotella* (5.80%) were identified as the major dominant genera. Prevotellaceae, Moraxellaceae, and Pasteurellaceae were among the dominant families in both lung and oral fluid samples. However, lung samples were distinct in having Enterobacteriaceae as the most abundant family (19.62%). At the genus level, lung and oral fluid diverged further in their dominant genera. Lung samples were dominated by a few genera, including *Escherichia–Shigella* (18.11%), *Actinobacillus* (14.45%), and *Mycoplasma* (7.05%). In contrast, oral fluid samples exhibited a more even distribution across multiple genera rather than being dominated by a few genera, with *Prevotella* (6.47%), *Moraxella* (5.86%), and *Streptococcus* (5.80%) as the most abundant.

To further identify taxa that significantly discriminated between lung and oral fluid samples, LEfSe analysis was conducted. A total of eight taxa with an LDA score ≥ 3 and a *p*-value < 0.05 were identified (Figure 1D). In lung samples, taxa such as *Escherichia–Shigella*, *Weissella*, and Yersiniaceae were relatively enriched, whereas oral fluid samples were enriched for taxa including *Moraxella*, *Streptococcus*, *Clostridium_sensu_stricto_1*, *Veillonella*, and *Bergeyella*.

### 3.3 Microbial community differences associated with pathogen status

To investigate microbial community differences according to pathogen detection status, alpha and beta diversity analyses were performed (Supplementary Table S5). Samples in this analysis were grouped based on pathogen positivity (positive vs. negative): oral fluid samples were classified according to the presence of respiratory pathogens, while fecal samples were grouped based on enteric pathogen status. Lung samples were excluded from this analysis due to limited sample size. Results demonstrating statistically significant differences in alpha and beta diversity by pathogen status are presented in Figure 2.

**Figure 2:**
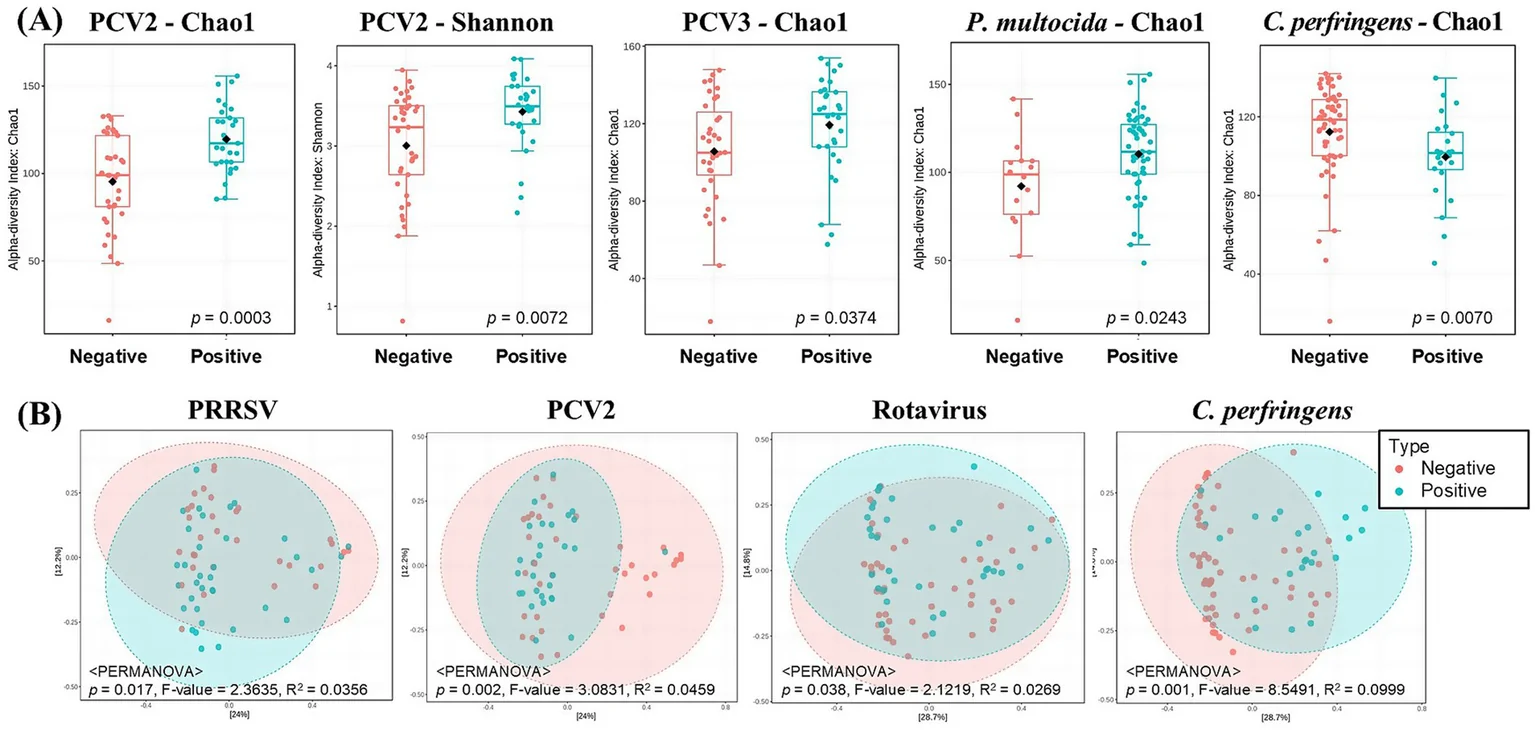
Alpha and beta diversity of microbial communities showing significant differences by pathogen status in oral fluid and fecal samples (*p* < 0.05). **(A)** Alpha diversity (Chao1 and Shannon indices) compared using the Mann–Whitney test. **(B)** Beta diversity visualized by PCoA based on Bray–Curtis dissimilarity.

Among the pathogens evaluated, *C. perfringens* was the only pathogen showing consistent associations with microbial diversity and community composition. In alpha diversity analysis (Figure 2A), the Chao1 index was significantly higher in the *C. perfringens*-negative group, indicating greater species richness compared to the positive group. Beta diversity analysis (Figure 2B) also revealed significant differences in microbial community structure between *C. perfringens*-positive and -negative groups (*p* < 0.05). Notably, no significant differences in dispersion were observed by PERMDISP (*p* > 0.05; Supplementary Table S5), indicating that the observed differences are unlikely to be driven by differences in group dispersion. Consistent with these findings, LEfSe analysis identified several taxa significantly associated with *C. perfringens* status (LDA score ≥ 4, *p* < 0.05). Specifically, *Bacteroides*, *Fusobacterium*, *Prevotella*, and Prevotellaceae_NK3B31_group were identified as key discriminative taxa (Figure 3A). *Bacteroides* and *Fusobacterium* were relatively enriched in the *C. perfringens*-positive group, whereas *Prevotella* and Prevotellaceae_NK3B31_group were significantly more abundant in the negative group.

**Figure 3:**
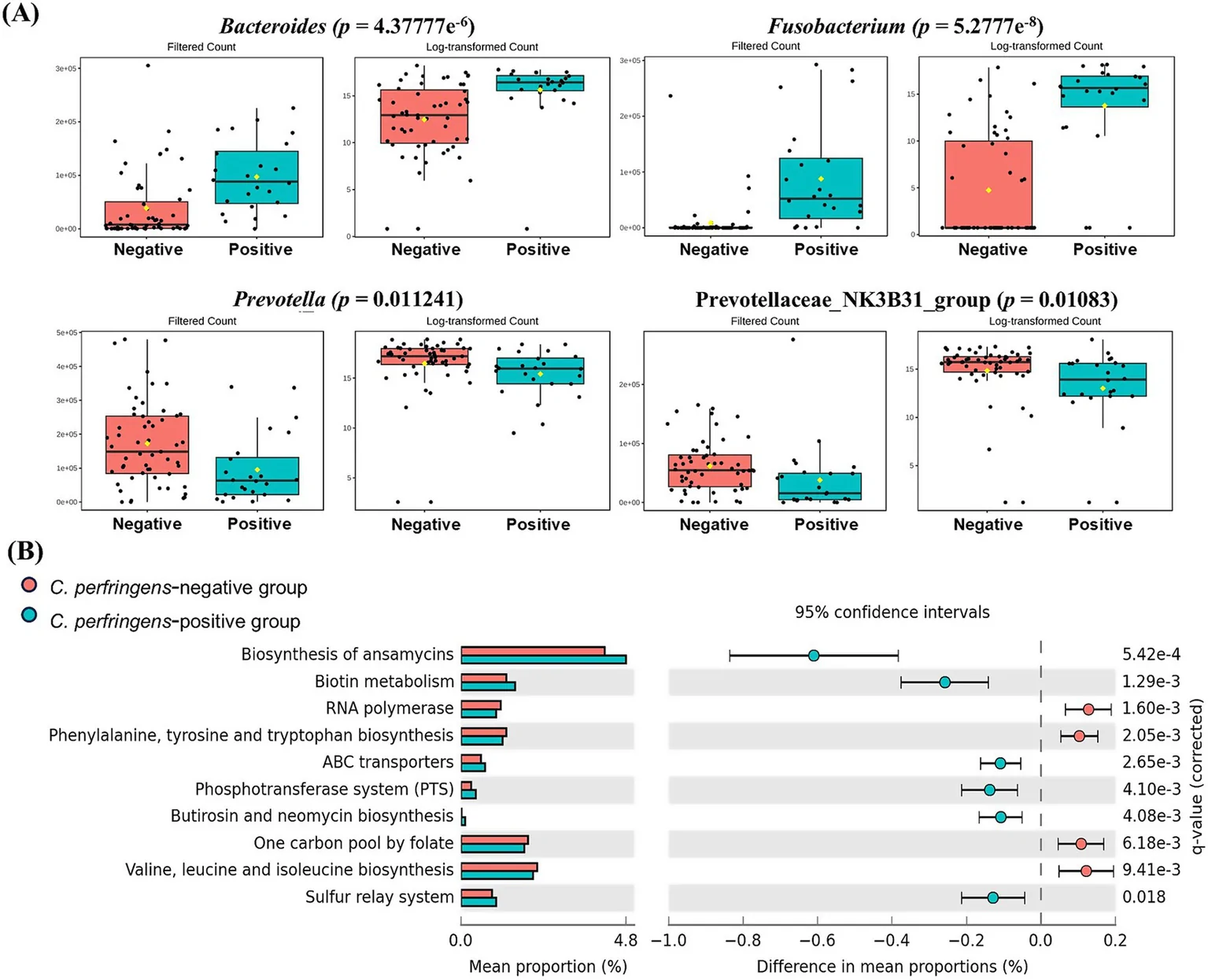
Taxonomic and predicted functional differences in fecal bacterial communities according to *Clostridium perfringens* status. **(A)** Filtered and log-transformed counts of the top four taxa identified by LEfSe as significantly associated with *Clostridium perfringens* status (LDA score ≥ 4 and *p* < 0.05). **(B)** Predicted functional profiles of bacterial communities according to *Clostridium perfringens* status. Functional profiles were predicted using PICRUSt2 and annotated based on the Kyoto Encyclopedia of Genes and Genomes (KEGG) database. Statistical significance was assessed using a two-sided Welch’s t-test, and only significantly different pathways are shown (*q* < 0.05). Bars represent the mean proportion (%) of each pathway, and the extended error bar plot shows the differences in mean proportions between groups with 95% confidence intervals.

To evaluate whether functional profiles differed according to *C. perfringens* detection status, PICRUSt2-based functional prediction was performed using 16S rRNA gene sequencing data (Figure 3B). Several predicted pathways differed significantly between the positive and negative groups. In the *C. perfringens*-positive group, biosynthesis of ansamycins, biotin metabolism, ABC transporters, phosphotransferase system (PTS), butirosin and neomycin biosynthesis and sulfur relay system pathways were predicted to be significantly more abundant compared to the negative group. In contrast, the predicted abundances of RNA polymerase; valine, leucine and isoleucine biosynthesis; one carbon pool by folate; and phenylalanine, tyrosine and tryptophan biosynthesis pathways were higher in the *C. perfringens*-negative group.

Beyond *C. perfringens*, other pathogens exhibited partial or statistically less robust associations with microbial diversity. In alpha diversity analysis (Figure 2A), Chao1 and Shannon indices were significantly higher in the PCV2-positive group. Similarly, the PCV3-positive and *P. multocida*-positive groups demonstrated higher species richness than the negative group. In beta diversity analysis (Figure 2B), significant differences in microbial community structure between pathogen-positive and -negative groups were observed for PRRSV, PCV2, and rotavirus (*p* < 0.05); however, PERMDISP analysis revealed significant dispersion differences for PRRSV and PCV2 (*p* < 0.05; Supplementary Table S5), suggesting that the observed PERMANOVA significance for these pathogens may have been influenced by within-group variability. For rotavirus, although PERMANOVA indicated a significant difference, subsequent LEfSe analysis did not identify any significantly discriminative taxa between positive and negative groups.

Sensitivity analyses accounting for farm-level clustering were performed for all comparisons by pathogen detection status (Supplementary Table S6). Among the significant differences described above, those associated with *C. perfringens* (Chao1, *p* = 0.017; PERMANOVA, *p* = 0.010), *P. multocida* (Chao1, *p* = 0.005), and rotavirus (PERMANOVA, *p* = 0.044) remained significant, whereas those associated with PCV2 (Chao1, Shannon, and PERMANOVA), PCV3 (Chao1), and PRRSV (PERMANOVA) were no longer significant. When the analyses were further restricted to farms that contributed both pathogen-positive and pathogen-negative samples, only the differences associated with *C. perfringens* remained significant (Chao1, *p* = 0.007; PERMANOVA, *p* = 0.017).

## 4 Discussion

In commercial swine production systems, identifying which pathogens are prevalent within farms is a key step for guiding preventive measures and protecting both animal health and farm productivity. In addition, the swine microbial community has been reported to be closely linked to host health through its roles in immune modulation and pathogen colonization (24, 25). Accordingly, microbiota characterization can contribute to a more comprehensive understanding of disease conditions. In this study, we investigated the detection rates of major respiratory and enteric pathogens in clinically symptomatic pigs from commercial swine farms in South Korea and characterized the microbial community structures of lung, oral fluid, and fecal samples. Furthermore, by combining field-based molecular diagnostics with microbiome profiling, we examined how microbial community structure varies according to pathogen detection status.

In this study, most respiratory pathogens showed higher detection rates in oral fluid than in lung samples. This pattern likely reflects sample-type characteristics: oral fluid represents a composite sample influenced by exploratory behaviors such as sniffing, licking, and ingestion (26). In addition, several pathogenic bacteria colonize the upper respiratory tract, including tonsils and nasal cavity, and may be carried asymptomatically (27). As oral fluid reflects herd-level pathogen circulation (26), the detection rates observed here suggest that diverse respiratory pathogens are widely circulating within the sampled farms. Among these pathogens, PRRSV was consistently detected at high rates in both lung (60.6%) and oral fluid (53.7%) samples. PRRSV is known for its efficient transmissibility and prolonged persistence: the virus is shed via nasal and oral secretions, urine, feces, and semen (28), and PCR positivity has been reported up to 157 days post-infection (29). Given this prolonged persistence, the high detection rate observed across lung and oral fluid samples may reflect both ongoing and past infections at individual and herd levels. However, it should be noted that detection itself does not establish active infection or lesion severity. Among enteric pathogens, rotavirus demonstrated the highest detection rate. According to previous large-scale studies in swine populations, including those from Spain and Denmark, rotavirus was identified as the most frequently detected pathogen associated with neonatal diarrhea (30, 31). Rotavirus infections are exacerbated by co-infection with other enteric pathogens, such as *E. coli*, *Clostridium* spp., and porcine epidemic diarrhea virus (PEDV), resulting in increased disease severity. The lack of specific antiviral treatments further complicates effective management (32). Notably, rotavirus A can cause acute gastroenteritis in both pigs and humans, and interspecies transmission has been documented, highlighting its zoonotic potential (33, 34). These findings support the continued monitoring of PRRSV and rotavirus in Korean commercial swine farms.

Distinct microbial community structures were observed among lung, oral fluid, and fecal samples. Interestingly, alpha diversity analysis showed that species diversity of oral fluid samples was comparable to that of fecal samples, which are generally considered to harbor highly diverse microbial communities. This finding suggests that oral fluid represents a matrix with relatively high microbial diversity. Beta diversity analysis further demonstrated clear separation with the greatest difference observed between respiratory samples (lung and oral fluid) and enteric samples (feces). The partial overlap between lung and oral fluid microbial communities may reflect their shared respiratory origin, whereas the clear separation of fecal samples likely reflects the distinct microbial ecology of the gastrointestinal environment.

In fecal samples, Firmicutes (40.63%) and Bacteroidota (42.97%) were identified as the dominant phyla, with the family Prevotellaceae characteristically enriched. Prevotellaceae has generally been regarded as a commensal taxon associated with intestinal homeostasis in healthy pigs (35, 36). However, some studies have also suggested potential associations between increased *Prevotella* abundance and diarrhea or intestinal inflammation (37, 38). These contrasting observations have been attributed to functional heterogeneity within Prevotellaceae at the species level, which may result in different associations depending on host immune and disease status. At the genus level, lung and oral fluid samples exhibited distinct microbial communities. Although *Escherichia–Shigella* is primarily associated with the gastrointestinal tract, it was identified as one of the predominant genera in lung samples (18.11%) and as a discriminative taxon in LEfSe analysis compared to oral fluid samples. Previous studies have also reported *Escherichia–Shigella* in the porcine lung microbiota regardless of disease status (39, 40). However, given the low biomass nature of lung specimens, the enrichment of *Escherichia–Shigella* and other lung-associated taxa identified in this study should be interpreted cautiously. Their detection may reflect true presence in the lower respiratory tract, aspiration or carryover from other anatomical sites, or technical or environmental contamination. In addition, oral fluid samples showed significantly higher relative abundances of *Moraxella* (5.86%) and *Streptococcus* (5.80%), which are commonly detected in the upper respiratory tract and oral mucosa of pigs. LEfSe analysis identified a greater number of discriminative taxa in oral fluid samples, including *Moraxella*, *Streptococcus*, *Clostridium_sensu_stricto_1*, *Veillonella*, and *Bergeyella*, compared to lung samples. These genera have been reported as core members of distinct mucosal niches in the porcine upper respiratory tract: *Moraxella* in the nasal cavity, *Veillonella* in the tonsils, and *Streptococcus* in the oropharynx (24, 41–43). Oral fluid is a composite sample that contacts the nasal cavity, tonsils, and oropharynx, which may account for the greater representation of taxa from these multiple niches compared with lung samples. LEfSe-identified taxa provide a microbial signature that clearly distinguishes lung and oral fluid samples, reflecting the distinct anatomical origins of the lower respiratory tract and the upper respiratory tract.

*C. perfringens* showed the most pronounced differences in microbial diversity and community composition according to its detection status in pig fecal microbiota. The *C. perfringens*-positive group showed significantly higher abundance of *Fusobacterium* and *Bacteroides*, whereas the negative group showed higher abundance of *Prevotella* and Prevotellaceae_NK3B31_group. *Fusobacterium* includes species such as *Fusobacterium nucleatum* and *Fusobacterium mortiferum* that have been associated with epithelial invasion and inflammatory bowel disease (IBD) in humans (44). The relatively higher abundance of *Fusobacterium* observed in the *C. perfringens*-positive group suggests that taxa with known associations to intestinal damage tend to co-occur in this microbial community context. However, a direct contribution to intestinal damage cannot be inferred from the present cross-sectional data, warranting further investigation. Although direct extrapolation requires caution due to host-specific differences, the inverse abundance pattern observed between *Bacteroides* and *Prevotella* in the present study parallels patterns documented in human intestinal disease states. Prior research has demonstrated that this inverse relationship is related to various intestinal disease conditions, including irritable bowel syndrome and colorectal cancer (45, 46).

PICRUSt2-based functional prediction further suggested contrasting metabolic potentials between the two groups. *C. perfringens*-positive microbiota showed relatively higher predicted abundances of pathways related to nutrient acquisition, environmental stress adaptation, and inter-microbial competition. In contrast, the negative group displayed higher predicted abundances of pathways related to cellular growth processes including transcription, amino acid biosynthesis, and one-carbon metabolism. These results suggest that the presence of *C. perfringens* may be associated with differences in both microbial composition and predicted functional profiles. To further elucidate these associations, future studies integrating multi-omics approaches will be necessary to clarify the functional mechanisms underlying these microbial alterations and assess their potential as biomarkers of *C. perfringens*-associated changes in the gut microbiota (47).

Several limitations should be acknowledged. As samples were submitted to a commercial veterinary diagnostic laboratory for routine diagnostic purposes, individual-level metadata (e.g., animal age, sex, diet, and disease severity) were unavailable. Although farm identifiers were available, unequal sampling across farms and multiple samples from the same farm raised the possibility of within-farm non-independence. In sensitivity analyses accounting for farm-level clustering, differences associated with *C. perfringens* remained significant under both farm inclusion criteria, whereas those associated with PCV2, PCV3, and PRRSV did not; differences associated with *P. multocida* and rotavirus were retained only when all farms were included. Thus, some pathogen-associated differences may have been influenced by shared farm-level factors, although the limited number of farms contributing both pathogen-positive and pathogen-negative samples may also have reduced statistical power. Finally, the absence of healthy control groups limited direct interpretation of disease-associated microbiota differences.

This study obtained diagnostic and microbiota data from clinically affected animals under natural field conditions. On commercial farms, pathogen diagnostic results are routinely used to assess the health status of animals. Because such results were available for each specimen in this study, microbial community composition could be interpreted alongside the diagnostic information. Most respiratory pathogens were detected more frequently in oral fluid than in lung samples, and the highest detection rates were observed for PRRSV in lung, *P. multocida* in oral fluid, and rotavirus in fecal samples. Microbiota analysis revealed distinct community structures among the three sample types. In particular, differences in gut microbial community composition and predicted functional profiles were observed according to the detection status of *C. perfringens*. This finding points to a possible association between pathogen detection and microbial community composition. Unlike controlled experimental infection models, the samples analyzed here reflect disease conditions in commercial production. These field-derived observations may serve as a reference for future research incorporating more comprehensive metadata.

## Data Availability

The 16S rRNA raw sequence datasets are available in the National Center for Biotechnology Information Sequence Read Archive repository (NCBI SRA) under accession number PRJNA1456896 (https://www.ncbi.nlm.nih.gov/bioproject/PRJNA1456896/). The deposited dataset comprises all 164 successfully sequenced samples analyzed in this study (lung, *n* = 19; oral fluid, *n* = 66; feces, *n* = 79). The data that support the findings of this study are available in the Supplementary material of this article.
