# Supplementary material for: Pathogen detection and microbial community characterization in clinically symptomatic pigs from commercial farms
Source: Front Vet Sci. 2026 Oct 1;13:1947134. doi: 10.3389/fvets.2026.1947134 (PMC13668500; doi:10.3389/fvets.2026.1947134)
Supplement: Supplementary file 1 [file Table_1.docx]

Supplementary Material

**Table S1.** Farm-level distribution of collected samples and samples included in microbiota analysis across 58 commercial pig farms.

| Farm | Samples collected | | | Samples included in microbiota analysis | | |
| --- | --- | --- | --- | --- | --- | --- |
|  | Lung | Oral fluid | Feces | Lung | Oral fluid | Feces |
| Farm 1 | 5 | 13 | 0 | 4 | 13 | 0 |
| Farm 2 | 1 | 9 | 5 | 0 | 9 | 5 |
| Farm 3 | 1 | 4 | 10 | 1 | 4 | 10 |
| Farm 4 | 0 | 7 | 0 | 0 | 6 | 0 |
| Farm 5 | 0 | 6 | 0 | 0 | 6 | 0 |
| Farm 6 | 0 | 0 | 6 | 0 | 0 | 6 |
| Farm 7 | 0 | 6 | 0 | 0 | 6 | 0 |
| Farm 8 | 1 | 1 | 3 | 0 | 1 | 3 |
| Farm 9 | 0 | 0 | 5 | 0 | 0 | 5 |
| Farm 10 | 0 | 5 | 0 | 0 | 5 | 0 |
| Farm 11 | 0 | 0 | 5 | 0 | 0 | 5 |
| Farm 12 | 0 | 0 | 4 | 0 | 0 | 4 |
| Farm 13 | 1 | 3 | 0 | 1 | 3 | 0 |
| Farm 14 | 0 | 0 | 4 | 0 | 0 | 4 |
| Farm 15 | 4 | 0 | 0 | 2 | 0 | 0 |
| Farm 16 | 0 | 0 | 3 | 0 | 0 | 3 |
| Farm 17 | 2 | 0 | 1 | 2 | 0 | 1 |
| Farm 18 | 0 | 3 | 0 | 0 | 3 | 0 |
| Farm 19 | 1 | 1 | 1 | 0 | 1 | 1 |
| Farm 20 | 1 | 0 | 2 | 1 | 0 | 2 |
| Farm 21 | 3 | 0 | 0 | 0 | 0 | 0 |
| Farm 22 | 0 | 3 | 0 | 0 | 3 | 0 |
| Farm 23 | 0 | 0 | 3 | 0 | 0 | 3 |
| Farm 24 | 2 | 0 | 0 | 0 | 0 | 0 |
| Farm 25 | 0 | 0 | 2 | 0 | 0 | 2 |
| Farm 26 | 0 | 0 | 2 | 0 | 0 | 2 |
| Farm 27 | 0 | 0 | 2 | 0 | 0 | 2 |
| Farm 28 | 0 | 0 | 2 | 0 | 0 | 2 |
| Farm 29 | 0 | 0 | 2 | 0 | 0 | 2 |
| Farm 30 | 0 | 0 | 2 | 0 | 0 | 2 |
| Farm 31 | 0 | 2 | 0 | 0 | 2 | 0 |
| Farm 32 | 0 | 1 | 1 | 0 | 1 | 1 |
| Farm 33 | 2 | 0 | 0 | 1 | 0 | 0 |
| Farm 34 | 0 | 1 | 1 | 0 | 1 | 1 |
| Farm 35 | 0 | 0 | 1 | 0 | 0 | 1 |
| Farm 36 | 0 | 0 | 1 | 0 | 0 | 1 |
| Farm 37 | 0 | 1 | 0 | 0 | 1 | 0 |
| Farm 38 | 0 | 0 | 1 | 0 | 0 | 1 |
| Farm 39 | 0 | 0 | 1 | 0 | 0 | 1 |
| Farm 40 | 1 | 0 | 0 | 1 | 0 | 0 |
| Farm 41 | 0 | 0 | 1 | 0 | 0 | 1 |
| Farm 42 | 0 | 0 | 1 | 0 | 0 | 1 |
| Farm 43 | 1 | 0 | 0 | 1 | 0 | 0 |
| Farm 44 | 0 | 0 | 1 | 0 | 0 | 1 |
| Farm 45 | 1 | 0 | 0 | 1 | 0 | 0 |
| Farm 46 | 1 | 0 | 0 | 1 | 0 | 0 |
| Farm 47 | 0 | 0 | 1 | 0 | 0 | 1 |
| Farm 48 | 0 | 0 | 1 | 0 | 0 | 1 |
| Farm 49 | 1 | 0 | 0 | 1 | 0 | 0 |
| Farm 50 | 0 | 1 | 0 | 0 | 1 | 0 |
| Farm 51 | 0 | 0 | 1 | 0 | 0 | 1 |
| Farm 52 | 0 | 0 | 1 | 0 | 0 | 1 |
| Farm 53 | 1 | 0 | 0 | 0 | 0 | 0 |
| Farm 54 | 0 | 0 | 1 | 0 | 0 | 1 |
| Farm 55 | 1 | 0 | 0 | 1 | 0 | 0 |
| Farm 56 | 0 | 0 | 1 | 0 | 0 | 1 |
| Farm 57 | 1 | 0 | 0 | 1 | 0 | 0 |
| Farm 58 | 1 | 0 | 0 | 0 | 0 | 0 |
| Total | 33 | 67 | 79 | 19 | 66 | 79 |

Note: “Samples included in microbiota analysis” refers to samples retained after 16S rRNA gene amplification and sequencing quality-control steps.

**Table S2.** Molecular diagnostic targets and methods for respiratory and enteric pathogens in lung, oral fluid, and fecal samples.

| Diagnostic sample | Diagnostic pathogen | | Target gene | Method |
| --- | --- | --- | --- | --- |
| Lung; Oral fluid | PRRSV (porcine reproductive and respiratory syndrome virus) | European (EU) | ORF6 | One step RT-qPCR |
|  |  | North American (NA) | ORF7, 3′UTR |  |
|  | PCV2 (porcine circovirus 2) | | ORF2 | Multiplex qPCR |
|  | PCV3 (porcine circovirus 3) | | ORF2 |  |
|  | *Actinobacillus pleuropneumoniae* | | *omlA* |  |
|  | *Pasteurella multocida* | | *kmt1* |  |
| Feces | Rotavirus | A | NSP3 | One step RT-qPCR |
|  |  | B | NSP5 |  |
|  |  | C | VP6 |  |
|  | Pathogenic *Escherichia coli* | F4 (K88) | *faeG* | Multiplex qPCR |
|  |  | F5 (K99) | K99 |  |
|  |  | F6 (987P) | 987P |  |
|  |  | F7 (F41) | F41 |  |
|  |  | F17 | *f17A* |  |
|  |  | F18 | *fedA* |  |
|  |  | LT | *eLTA* |  |
|  |  | STa | *Sta1* |  |
|  |  | STb | *Stb1* |  |
|  |  | Stx 2e | *Stx2e* |  |
|  | *Clostridium perfringens* | CPA | *cpa* |  |
|  |  | CPB | *cpb* |  |
|  |  | CPB2 | *cpb2* |  |
|  |  | CPE | *cpe* |  |

Note: PRRSV positivity was defined as detection of either the EU or NA type. Pathogenic *E. coli* positivity was defined as detection of at least one fimbrial antigen (F4, F5, F6, F7, F17, F18) and at least one toxin (LT, STa, STb, Stx 2e). Rotavirus positivity was defined as detection of any of the A, B, or C species. *C. perfringens* positivity was defined as detection of at least one toxin gene (CPA, CPB, CPB2, CPE).

**Table S3.** Pairwise associations between pathogens within each sample type based on odds ratios and Fisher’s exact test.

| Pathogen1 (P1) | Pathogen2 (P2) | OR | 95% CI | Unadjusted *p*-value | *q*-value |
| --- | --- | --- | --- | --- | --- |
| Lung (*n* = 33) | | | | | |
| PRRSV | PCV2 | 6.336 | 0.99-73.39 | 0.032 | 0.219 |
| PRRSV | PCV3 | Inf | 0.65-Inf | 0.131 | 0.295 |
| PRRSV | PM | Inf | 0.87-Inf | 0.060 | 0.219 |
| PRRSV | APP | 0.641 | 0.01-53.64 | 1.000 | 1.000 |
| PCV2 | PCV3 | 7.887 | 0.66-435.90 | 0.066 | 0.219 |
| PCV2 | PM | 3.823 | 0.45-49.90 | 0.182 | 0.304 |
| PCV2 | APP | Inf | 0.30-Inf | 0.148 | 0.295 |
| PCV3 | PM | 3.783 | 0.25-46.43 | 0.216 | 0.309 |
| PCV3 | APP | 0 | 0.00-32.03 | 1.000 | 1.000 |
| PM | APP | 4.837 | 0.06-421.66 | 0.335 | 0.419 |
| Oral fluid (*n* = 67) | | | | | |
| PRRSV | PCV2 | 1.409 | 0.48-4.22 | 0.622 | 0.777 |
| PRRSV | PCV3 | 0.955 | 0.33-2.78 | 1.000 | 1.000 |
| PRRSV | PM | 1.681 | 0.47-6.24 | 0.401 | 0.668 |
| PRRSV | APP | 0.353 | 0.10-1.12 | 0.068 | 0.226 |
| PCV2 | PCV3 | 3.579 | 1.18-11.49 | 0.014 | 0.072 |
| PCV2 | PM | 1.937 | 0.53-8.18 | 0.387 | 0.668 |
| PCV2 | APP | 0.867 | 0.27-2.73 | 1.000 | 1.000 |
| PCV3 | PM | 2.442 | 0.66-10.32 | 0.159 | 0.398 |
| PCV3 | APP | 1.491 | 0.48-4.75 | 0.603 | 0.777 |
| PM | APP | 10.23 | 1.37-461.63 | 0.013 | 0.072 |
| Fecal (*n* = 79) | | | | | |
| Rotavirus | *E. coli* | 3.419 | 1.21-10.14 | 0.018 | 0.053 |
| Rotavirus | *C. perfringens* | 1.897 | 0.65-5.66 | 0.222 | 0.222 |
| *E. coli* | *C. perfringens* | 2.211 | 0.74-6.68 | 0.131 | 0.197 |

Note: OR, odds ratio; *q*-value, FDR-adjusted *p*-value calculated using the Benjamini–Hochberg procedure; Inf, infinity; PM, *Pasteurella multocida*; APP, *Actinobacillus pleuropneumoniae.* Infinite odds ratios indicate that one of the discordant outcome categories was not observed.

**Table S4**. Pairwise comparisons of microbial community composition between sample types based on Bray–Curtis dissimilarity (PERMANOVA).

| Group 1 | Group 2 | *p*-value | F-value | R^2^ |
| --- | --- | --- | --- | --- |
| Lung | Oral fluid | 0.001 | 10.278 | 0.11018 |
| Lung | Feces | 0.001 | 18.045 | 0.15823 |
| Oral fluid | Feces | 0.001 | 26.917 | 0.15841 |

**Table S5.** Microbial diversity analyses by diagnostic pathogen status (positive vs. negative groups) in oral fluid and fecal samples.

| Diagnostic pathogen | Alpha diversity | | PERMANOVA | | | PERMDISP | |
| --- | --- | --- | --- | --- | --- | --- | --- |
|  | Chao1 *p*-value | Shannon *p*-value | *p*-value | F-value | R^2^ | *p*-value | F-value |
| PRRSV | 0.0809 | 0.0905 | *0.017 | 2.3635 | 0.0356 | *0.0032 | 9.3959 |
| PCV2 | **0.0003 | *0.0072 | *0.002 | 3.0831 | 0.0459 | *0.0072 | 7.7150 |
| PCV3 | *0.0374 | 0.1785 | 0.468 | 0.9521 | 0.0147 | 0.1394 | 2.2394 |
| *P. multocida* | *0.0243 | 0.5346 | 0.053 | 1.7462 | 0.0266 | 0.8520 | 0.0351 |
| *A. pleuropneumoniae* | 0.8490 | 0.9946 | 0.122 | 1.4163 | 0.0216 | 0.7922 | 0.0699 |
| Pathogenic *E. coli* | 0.2716 | 0.2816 | 0.071 | 1.8039 | 0.0229 | 0.1339 | 2.2943 |
| Rotavirus | 0.2696 | 0.2304 | *0.038 | 2.1219 | 0.0269 | 0.2279 | 1.4772 |
| *C. perfringens* | *0.0070 | 0.0742 | **0.001 | 8.5491 | 0.0999 | 0.7163 | 0.1330 |

Note: Pairwise differences were evaluated by pathogen status (positive vs. negative) in oral fluid samples for respiratory pathogens and in fecal samples for enteric pathogens. Alpha diversity (Chao1 and Shannon indices) was assessed using the Mann–Whitney test. Beta diversity was evaluated using Bray–Curtis dissimilarity, followed by PERMANOVA and PERMDISP analyses. **p* < 0.05; ***p* < 0.001.

**Table S6.** Sensitivity analyses of microbial diversity by diagnostic pathogen status (positive vs. negative groups) in oral fluid and fecal samples, accounting for farm-level clustering.

| **Diagnostic pathogen** | **Analysis** | **Restricted-permutation *p*-value** | |
| --- | --- | --- | --- |
|  |  | **All farms** | **Farms with both statuses** |
| PRRSV | Chao1 | 0.191 | 0.812 |
|  | Shannon | 0.275 | 0.436 |
|  | Beta diversity | 0.065 | 0.172 |
| PCV2 | Chao1 | 0.076 | 0.104 |
|  | Shannon | 0.159 | 0.222 |
|  | Beta diversity | 0.195 | 0.293 |
| PCV3 | Chao1 | 0.064 | 0.062 |
|  | Shannon | 0.184 | 0.213 |
|  | Beta diversity | 0.526 | 0.493 |
| *P. multocida* | Chao1 | *0.005 | 0.135 |
|  | Shannon | 0.323 | 0.950 |
|  | Beta diversity | *0.014 | 0.050 |
| *A. pleuropneumoniae* | Chao1 | 0.409 | 0.396 |
|  | Shannon | 0.987 | 0.300 |
|  | Beta diversity | 0.515 | 0.253 |
| Pathogenic *E. coli* | Chao1 | 0.442 | 0.551 |
|  | Shannon | 0.484 | 0.615 |
|  | Beta diversity | 0.551 | 0.547 |
| Rotavirus | Chao1 | 0.167 | 0.160 |
|  | Shannon | 0.366 | 0.943 |
|  | Beta diversity | *0.044 | 0.405 |
| *C. perfringens* | Chao1 | *0.017 | *0.007 |
|  | Shannon | 0.392 | 0.398 |
|  | Beta diversity | *0.010 | *0.017 |

Note: Alpha diversity was evaluated using the Mann–Whitney U statistic and beta diversity using PERMANOVA based on Bray–Curtis dissimilarity, with 999 permutations restricted within farms. "All farms" includes all sampled farms, whereas "Farms with both statuses" includes only farms with at least one pathogen-positive and one pathogen-negative sample. Because these analyses were conducted as sensitivity analyses of the primary comparisons, the permutation-derived *p*-values were not subjected to additional multiplicity adjustment.**p* < 0.05.
